# Healthcare for individuals with schizophrenia in Taiwan: 10-year national trend analysis

**DOI:** 10.1192/bjo.2025.10868

**Published:** 2025-10-13

**Authors:** Shen-Yu Tsai, Ming-Shiang Wu, Shi-Heng Wang, Shih-Cheng Liao, Wei J Chen, Chi-Shin Wu

**Affiliations:** Attending Physician, Department of Psychiatry, National Taiwan University Hospital, Hsin-Chu Branch, Hsinchu, Taiwan; Data analyst, National Center for Geriatrics and Welfare Research, National Health Research Institutes, Miaoli, Taiwan; Associate Professor, Department of Medical Research, China Medical University Hospital, China Medical University, Taichung, Taiwan; Professor, Department of Psychiatry, College of Medicine, National Taiwan University, Taipei, Taiwan; Attending Physician, Department of Psychiatry, National Taiwan University Hospital, Taipei, Taiwan; Distinguished Investigator, Center for Neuropsychiatric Research, National Health Research Institutes, Miaoli, Taiwan; Distinguished Professor, Institute of Epidemiology and Preventive Medicine, College of Public Health, National Taiwan University, Taipei, Taiwan; Adjunct Attending Physician, Department of Psychiatry, National Taiwan University Hospital, Yunlin Branch, Yunlin, Taiwan; Investigator, National Center for Geriatrics and Welfare Research, National Health Research Institutes, Miaoli, Taiwan

**Keywords:** Schizophrenia, quality of healthcare, antipsychotic agents, hospitalisation, employment

## Abstract

**Background:**

Significant changes in Taiwan’s psychiatric services over recent decades include expansion of community-based clinics and implementation of the Schizophrenia Pay-for-Performance programme.

**Aims:**

This study aimed to assess the trend of the quality of healthcare for individuals with schizophrenia, using various indicators of the treatment process and outcomes between 2010 and 2019.

**Method:**

Individuals with schizophrenia were identified using Taiwan’s National Health Insurance claims database. The quality of healthcare for individuals with schizophrenia was assessed using treatment process and outcome indicators, including antipsychotic types, medication adherence, daily dose for antipsychotics and concurrent use of other psychotropic agents. Outcome indicators included all-cause mortality, suicide deaths, psychiatric hospitalisation, emergency department visits and employment status.

**Results:**

Antipsychotic medication usage has shifted towards second-generation antipsychotics (SGAs) and long-acting injectable antipsychotics (LAIs), with declines in first-generation antipsychotics. The percentage of medication adherence declined, while that of individuals with an adequate daily dose increased. Concurrently, anticholinergic and benzodiazepine use decreased while antidepressant and mood stabiliser use increased. Outcome indicators showed no significant change in all-cause mortality or suicide rates over time, but there were reductions in psychiatric hospitalisations and emergency department visits. Employment rates increased overall, particularly in urban areas.

**Conclusions:**

The quality of healthcare for individuals with schizophrenia, as measured by treatment process and outcome indicators, improved alongside changes in Taiwan’s psychiatric services; however, causality cannot be inferred from our findings. Future research should evaluate the effectiveness of psychiatric service policies and continuously monitor healthcare quality to further enhance the lives of individuals with schizophrenia.

Schizophrenia is a severe mental disorder with a relapsing course. Only a small fraction of individuals with schizophrenia – less than 15% – achieve sustained recovery, while the majority experience recurrent relapses.^1^ Moreover, only a minority are able to maintain employment.^2^ Schizophrenia is also associated with markedly higher mortality: individuals with schizophrenia have a two- to threefold higher risk of death.^3^ Among the causes of death, suicide accounts for approximately 28% of excess mortality in schizophrenia.^4^ Adherence to antipsychotic treatment is a vital component of care, because discontinuation of medication significantly increases the risk of relapse and hospitalisation^5^ and even elevates the risk of death after a first episode.^6^ Long-acting injectable antipsychotics (LAIs) have been introduced to improve adherence. Compared with oral medications, LAIs can reduce all-cause treatment discontinuation, hospitalisations and readmissions,^7,8^ and may also lower all-cause mortality and suicide rates.^9^ Given these advantages, many countries have widely adopted LAIs in routine care.

Significant transformations have reshaped psychiatric services in Taiwan. In 1996, the universal National Health Insurance (NHI) programme was introduced, enhancing overall healthcare access. A study from 1998 to 2007 showed reduced readmission and 30-day dropout rates for in-patient schizophrenia, although rural areas saw slower improvements, widening urban–rural disparities.^10^ Over the past decade, the expansion of community-based psychiatric clinics has further improved mental healthcare accessibility.^11^ Additionally, the Schizophrenia Pay-for-Performance (P4P) programme, launched in January 2010, aims to elevate care quality at psychiatric facilities. The programme comprises four components: (a) establishing a treatment plan and goals; (b) promoting multidisciplinary care teams to provide self-management education and consultation; (c) assigning a case manager to support follow-up and encourage regular visits; and (d) offering financial incentives to reward improved facility performance, especially for patients who infrequently visit or have never visited.^12^ All facilities offering psychiatric services are eligible to apply for participation. Recent enhancements to the programme, such as LAI administration and community visits, require further study to assess their impact on the quality of healthcare for patients with schizophrenia.

These policies may have an impact on the overall quality of care for schizophrenia in Taiwan, but further research is needed. We have analysed data from 2010 to 2019, examining indicators related to the treatment process and outcomes.

## Method

### Data source

This study utilised a claims database obtained from Taiwan’s NHI programme, which had an enrolment of approximately 23.0 million individuals and a coverage rate of 99% as of 2009. The claims database contained comprehensive information on demographic variables, prescription records and clinical diagnoses. Prior to 2016, diagnoses were coded according to ICD-9, subsequently updated to ICD-10 from 2016 onwards. Previous research has extensively documented the accuracy of diagnostic codes for major psychiatric disorders.^13^ This study was approved by the Research Ethics Committee of the National Taiwan University Hospital (no. 201204034RIC). The NHI claims that data were fully deidentified, and all analyses were conducted in accordance with relevant privacy and confidentiality guidelines; informed consent was therefore waived.

### Study sample

The study’s participant pool encompassed all individuals diagnosed with schizophrenia-spectrum disorders, which include both schizophrenia and schizoaffective disorders. We identified these cases using ICD codes, specifically ICD-9-CM: 295 and ICD-10: F20 and F25. These codes were extracted from records spanning out-patient, emergency department and in-patient claims records, covering the period 2010 to 2019. To ensure the accuracy of our diagnoses, we included only individuals who had been diagnosed or treated by psychiatrists. We evaluated the number of schizophrenia cases and pertinent demographic factors such as age, gender and urban residency status for each calendar year. The prevalence of schizophrenia was determined by case numbers and percentages, using mid-year population counts as denominators obtained from Taiwan’s National Statistics. Additionally, we estimated the prevalence stratified by key demographic variables, including age groups, gender and urbanicity.

### Quality indicator for healthcare of individuals with schizophrenia

The evaluation of medical care quality included process and outcome indicators,^14^ measured annually. This evaluation assessed medication types – first-generation antipsychotics (FGAs), second-generation antipsychotics (SGAs) and LAIs – and the adequacy of treatment. LAIs were further classified into FGA LAIs and SGA LAIs. The use of other psychotropic drugs, such as antidepressants and mood stabilisers, was also examined. In terms of treatment adequacy, the medication adherence of antipsychotic use was evaluated using the medication possession ratio (MPR), calculated as the total days’ supply of antipsychotics per year divided by 365. Because the days’ supply for LAI antipsychotics (LAIs) may be inaccurate in the NHI claims database, we defined this for LAIs as follows: 14 days for risperidone; 21 days for flupentixol decanoate, fluphenazine decanoate and zuclopentixol decanoate; 28 days for aripiprazole monohydrate, haloperidol decanoate and paliperidone palmitate (INVEGA SUSTENNA®); and 90 days for paliperidone palmitate (INVEGA TRINZA®).^15^ We defined medication adherence as an MPR of 0.8 or higher which, as a threshold, is a commonly accepted standard to denote adequate adherence.^16^ Due to potential inaccuracies in the days’ supply and lack of dose information, the average daily dose was used to assess antipsychotic use, converting doses into defined daily doses (DDDs), a statistical measure used by the World Health Organization.^17^ The total DDD per year was divided by 365 to determine the average daily dose, with values ≥0.8 considered adequate. We selected the 0.8 cut-off for consistency with established practices in evaluating adherence and dosing adequacy.^16^

The outcome indicators considered in this study encompassed various events related to schizophrenia-spectrum disorders, including all-cause mortality, suicide deaths, psychiatric hospitalisation, emergency department visits and employment status. Dates of death were obtained from Taiwan’s National Death Registry, and suicide cases were identified based on ICD-9-CM external codes E950–9 or ICD-10 codes X60–84 or X87.0. Emergency department visits and psychiatric hospitalisations were tracked using a binary variable that flagged any psychiatric-related visits (ICD-9: 290–319 or ICD-10: F00–99), or admissions to psychiatric wards or hospitals within the year. Employment status was assessed by the Registry for NHI beneficiaries, which provided occupation categories and identifiers for insured individuals and income earners. Employment was determined monthly; individuals were categorised as employed if they held a job at any point during the month.

### Statistical analysis

The characteristics of individuals with schizophrenia were described, including their age group (0–14, 15–24, 25–34, 35–44, 45–54, 55–64 and ≥65 years), gender and urbanicity of their living area (urban/rural). The degree of urbanisation was assessed using a cluster analysis that considered five key factors: population density, physician density, the percentage of individuals with a college education or higher, the proportion of senior citizens and the share of the workforce engaged in agriculture.^18^ Each township was categorised into seven levels, and further dichotomised into either urban (urbanicity levels 1–3) or rural (urbanicity levels 4–7).

To investigate temporal trends in the treatment process and clinical outcome indicators among individuals with schizophrenia, the calendar year was standardised by subtracting 2010 from the year and dividing the result by 9. Consequently, the standardised values represented 0 for 2010 and 1 for 2019.^19^ These transformed values of the calendar year allowed for assessment of changes in healthcare quality indicators for individuals with schizophrenia throughout the entire study period from 2010 to 2019. Adjusted odds ratios (AORs) were calculated using a multivariate logistic model, which accounted for changes in age groups, gender and urbanicity over the study duration, because these factors influenced both treatment patterns and outcomes. To explore urban–rural disparities, subgroup analyses based on urbanisation were conducted.

The analyses were performed using SAS version 9.4 for Windows (SAS Institute Inc., Cary, NC, USA; see https://www.sas.com/en_us/software/stat.html), with a significance level of *P* < 0.05 considered statistically significant for all tests.

## Results

During the study period from 2010 to 2019 the prevalence of schizophrenia in Taiwan increased slightly, from 98 733 cases in 2010 to 105 568 in 2019, and the percentage rose from 0.42 to 0.46. The mean age of individuals diagnosed with schizophrenia also increased, from 43.7 years at the beginning of the study to 47.7 years by the end. This prevalence rose among individuals aged 45 or older, while it declined for those under 44. A significant increase was observed in rural areas, rising from 0.46 to 0.51%. In contrast, urban areas experienced a smaller increase, from 0.41 to 0.43% (see Table 1). With the large sample size, all observed trends reached statistical significance (*P* < 0.01).

**Table 1:** Characteristics of schizophrenia patients, from 2010 to 2019

Characteristic	2010	2011	2012	2013	2014	2015	2016	2017	2018	2019
Overall, prevalence (%)^a^	98 733 (0.42)	99 896 (0.42)	10, 969 (0.43)	101 447 (0.43)	102 477 (0.44)	104 199 (0.44)	105 323 (0.45)	105 481 (0.45)	106 072 (0.46)	105 568 (0.46)
Age groups, years										
0–14	222 (0.01)	186 (0.01)	144 (0)	139 (0)	150 (0)	139 (0)	133 (0)	133 (0)	114 (0)	105 (0)
15–24	5585 (0.20)	5410 (0.19)	5234 (0.18)	4914 (0.16)	4817 (0.16)	4667 (0.15)	4567 (0.14)	4580 (0.14)	4473 (0.14)	4334 (0.14)
25–34	19 180 (0.60)	18 238 (0.56)	17 385 (0.53)	16 456 (0.49)	15 634 (0.45)	14 843 (0.42)	14 132 (0.39)	13 598 (0.36)	13 050 (0.34)	12 518 (0.32)
35–44	27 977 (0.71)	27 939 (0.71)	27 827 (0.71)	27 640 (0.72)	27 456 (0.72)	27 486 (0.73)	27 490 (0.74)	27 054 (0.73)	26 584 (0.72)	25 697 (0.70)
45–54	26 400 (0.74)	27 420 (0.76)	28 242 (0.77)	28 669 (0.78)	29 224 (0.79)	29 948 (0.81)	30 260 (0.81)	29 956 (0.81)	30 000 (0.81)	29 820 (0.81)
55–64	14 751 (0.42)	15 876 (0.46)	16 990 (0.50)	18 267 (0.55)	19 293 (0.59)	20 628 (0.65)	21 663 (0.71)	22 556 (0.77)	23 549 (0.85)	24 272 (0.93)
≥65	4618 (0.13)	4827 (0.14)	5147 (0.16)	5362 (0.17)	5903 (0.20)	6488 (0.23)	7078 (0.26)	7604 (0.29)	8302 (0.33)	8822 (0.35)
Age, mean ± s.d.	43.6 ± 12.5	44.1 ± 12.5	44.6 ± 12.5	45.1 ± 12.5	45.6 ± 12.5	46.1 ± 12.5	46.5 ± 12.6	46.8 ± 12.7	47.3 ± 12.7	47.7 ± 12.7
Gender										
Female	46 719 (0.40)	47 567 (0.41)	48 307 (0.41)	48 708 (0.42)	49 365 (0.42)	50 489 (0.43)	50 969 (0.44)	51 198 (0.44)	5, 807 (0.44)	51 641 (0.44)
Male	52 014 (0.44)	52 329 (0.44)	52 662 (0.44)	52 739 (0.45)	53 112 (0.45)	53 710 (0.46)	54 354 (0.47)	54 283 (0.47)	54 265 (0.47)	53 927 (0.47)
Urbanisation										
Urban	69 103 (0.41)	69 998 (0.42)	70 919 (0.42)	71 147 (0.42)	72 183 (0.42)	73 419 (0.42)	74 290 (0.43)	74 475 (0.43)	74 907 (0.43)	74 766 (0.43)
Rural	29 630 (0.46)	29 898 (0.47)	30 050 (0.47)	30 300 (0.48)	30 294 (0.49)	30 780 (0.50)	31 033 (0.51)	31 006 (0.51)	31 165 (0.51)	30 802 (0.51)

a. The population denominators for each year are the number of individuals in mid-year, from Taiwan’s National Statistics.

In pharmacological treatment trends, there has been a shift in antipsychotic medication use over the decade under study. FGA use decreased from 23.1 to 16.0%, while that of SGA increased from 79.0 to 82.9%. LAI use rose from 11.8 to 16.1%, notably with a decline in FGA LAIs and a surge in SGA LAIs. Medication adherence decreased from 38.3 to 34.0%, corresponding to a decrease of 0.82-fold (95% CI: 0.81–0.83). Adequate daily dose increased from 21.0 to 27.2%, indicating a rise of 1.44-fold (95% CI: 1.42–1.46). Concurrent usage of other psychotropic agents showed declines in anticholinergics and benzodiazepines but increases in antidepressants and mood stabilisers (see Table 2 for details).

**Table 2:** Ten-year trends in treatment process indicators for schizophrenia patients in Taiwan, stratified by urban and rural areas

Characteristic	2010	2011	2012	2013	2014	2015	2016	2017	2018	2019	Crude odds ratio (95% CI)	Adjusted odds ratio (95% CI)
Overall (%)												
Antipsychotic types												
FGA	23.1	22.3	21.3	20.5	19.9	18.8	17.5	17.0	16.5	16.0	0.62 (0.61, 0.63)	0.60 (0.59, 0.61)
SGA	79.0	80.0	81.0	81.7	82.1	82.4	82.3	82.6	82.5	82.9	1.26 (1.24, 1.28)	1.37 (1.35, 1.39)
LAI	11.8	11.7	11.7	11.8	12.2	12.5	12.8	13.5	14.6	16.1	1.43 (1.40, 1.45)	1.46 (1.44, 1.49)
FGA LAI	9.2	8.9	8.7	8.4	8.5	8.3	7.9	7.7	7.5	7.4	0.79 (0.77, 0.81)	0.78 (0.77, 0.80)
SGA LAI	3.1	3.2	3.5	3.9	4.1	4.7	5.5	6.5	7.9	9.5	3.54 (3.44, 3.64)	3.83 (3.72, 3.94)
Treatment adequacy												
Good adherence, MPR ≥ 0.8	38.3	37.6	37.0	37.3	36.8	36.3	35.2	35.1	34.6	34.0	0.84 (0.83, 0.85)	0.82 (0.81, 0.83)
Adequate daily dose, DDD ≥ 0.8	21.0	22.1	23.0	23.8	24.2	24.6	25.5	26.2	26.8	27.2	1.39 (1.37, 1.41)	1.44 (1.42, 1.46)
Other psychotropic agents												
Antidepressant	23.9	24.2	24.6	24.9	25.0	25.3	25.5	26.0	26.5	27.2	1.17 (1.16, 1.19)	1.23 (1.21, 1.25)
BZD	72.3	72.3	72.0	71.4	70.7	70.0	69.4	69.1	68.4	68.6	0.81 (0.80, 0.82)	0.80 (0.79, 0.81)
Anticholinergic	53.4	52.5	51.6	50.6	49.4	48.0	46.8	46.2	45.1	44.6	0.69 (0.68, 0.70)	0.67 (0.67, 0.68)
Mood stabiliser	15.9	16.0	15.9	16.0	16.1	16.1	16.2	16.4	16.6	16.9	1.07 (1.05, 1.09)	1.13 (1.11, 1.15)
Urban (%)												
Antipsychotic types												
FGA	22.7	21.8	20.9	20.1	19.5	18.6	17.3	16.7	16.2	15.7	0.62 (0.61, 0.64)	0.60 (0.59, 0.62)
SGA	79.6	80.7	81.5	82.3	82.6	82.9	82.6	82.9	82.9	83.2	1.23 (1.21, 1.25)*	1.34 (1.32, 1.37)*
LAI	11.8	11.7	11.8	11.9	12.2	12.5	12.8	13.5	14.7	16.2	1.42 (1.39, 1.45)	1.46 (1.42, 1.49)
FGA LAI	9.0	8.7	8.6	8.4	8.5	8.2	7.7	7.6	7.4	7.3	0.80 (0.77, 0.82)	0.78 (0.76, 0.81)
SGA LAI	3.4	3.5	3.8	4.0	4.3	4.9	5.6	6.6	8.1	9.6	3.32 (3.21, 3.43)*	3.58 (3.46, 3.70)*
Treatment adequacy												
Good adherence, MPR ≥ 0.8	37.9	37.2	36.6	36.8	36.2	35.8	34.6	34.6	34.0	33.3	0.90 (0.88, 0.91)*	0.89 (0.88, 0.90)*
Adequate daily dose, DDD ≥ 0.8	31.1	31.9	33.3	33.9	34.1	34.4	35.2	35.7	36.3	36.8	1.27 (1.25, 1.29)*	1.31 (1.29, 1.33)*
Other psychotropic agens												
Antidepressant	24.8	25.2	25.3	25.7	25.7	26.2	26.3	26.7	27.3	28.1	1.16 (1.15, 1.18)	1.22 (1.20, 1.24)
BZD	71.7	71.7	71.4	70.9	70.1	69.7	68.9	68.8	68.1	68.2	0.82 (0.81, 0.83)*	0.81 (0.80, 0.82)*
Anticholinergic	52.4	51.6	50.6	49.5	48.3	46.9	45.7	45.3	44.2	43.7	0.69 (0.68, 0.70)	0.67 (0.66, 0.68)
Mood stabiliser	15.5	15.6	15.5	15.6	15.6	15.6	15.7	15.9	16.1	16.4	1.06 (1.04, 1.08)	1.12 (1.09, 1.14)
Rural (%)												
Antipsychotic types												
FGA	24.0	23.3	22.3	21.7	20.7	19.4	18.0	17.6	17.3	16.8	0.62 (0.60, 0.63)	0.61 (0.59, 0.62)
SGA	77.5	78.4	79.7	80.1	80.9	81.2	81.5	81.9	81.5	82.3	1.32 (1.29, 1.36)*	1.42 (1.38, 1.47)*
LAI	11.7	11.5	11.4	11.6	12.0	12.4	12.8	13.5	14.5	15.9	1.43 (1.39, 1.48)	1.48 (1.43, 1.54)
FGA LAI	9.6	9.2	8.7	8.6	8.6	8.5	8.2	7.9	7.6	7.5	0.78 (0.75, 0.81)	0.78 (0.75, 0.82)
SGA LAI	2.6	2.7	3.0	3.4	3.7	4.5	5.2	6.2	7.6	9.2	4.19 (3.97, 4.43)*	4.54 (4.30, 4.80)*
Treatment adequacy												
Good adherence, MPR ≥ 0.8	39.0	38.7	38.0	38.5	38.1	37.4	36.6	36.3	36.2	35.7	0.95 (0.93, 0.97)*	0.93 (0.91, 0.95)*
Adequate daily dose, DDD ≥ 0.8	20.2	21.7	22.4	22.9	23.5	24.3	25.3	26.1	26.7	27.3	1.37 (1.34, 1.40)*	1.43 (1.39, 1.46)*
Other psychotropic agents												
Antidepressant	21.6	22.0	22.8	23.1	23.2	23.2	23.5	24.1	24.5	25.2	1.19 (1.16, 1.22)	1.25 (1.22, 1.28)
BZD	73.8	73.6	73.4	72.7	72.0	70.7	70.4	70.0	69.3	69.7	0.78 (0.76, 0.80)*	0.78 (0.76, 0.80)*
Anticholinergic	55.6	54.6	53.8	53.2	52.1	50.5	49.4	48.3	47.4	46.9	0.69 (0.68, 0.71)	0.68 (0.66, 0.70)
Mood stabiliser	16.7	17.1	17.0	17.0	17.3	17.2	17.4	17.7	18.0	18.1	1.09 (1.06, 1.13)	1.16 (1.12, 1.19)

BZD, benzodiazepine; DDD, defined daily dose; FGA, first-generation antipsychotic; LAI, long-acting injectable antipsychotic; MPR, medication possession ratio; SGA, second-generation antipsychotic.

Adjusted odds ratios were calculated using a multivariate logistic model, which accounted for changes in age groups, gender and urbanicity.

*Significant urban–rural difference (*P* < 0.05).

Regarding outcome indicators, our investigation found no significant shifts in trends for all-cause mortality and suicide mortality. However, there was a decrease in the percentages of psychiatric hospitalisations and emergency department visits, showing reductions of 0.91- and 0.97-fold, respectively, over the decade. Notably, employment rates initially showed no significant change, ranging from 36.8% in 2010 to 36.1% in 2019. However, after accounting for demographic variables, we observed a slight increase of 1.05-fold (see Table 3).

**Table 3:** Ten-year trends in outcome indicators for schizophrenia patients in Taiwan, stratified by urban and rural areas

Characteristic	2010	2011	2012	2013	2014	2015	2016	2017	2018	2019	Crude odds ratio (95% CI)	Adjusted odds ratio (95% CI)
Overall (%)												
All-cause mortality	1.04	1.13	1.14	1.18	1.22	1.23	1.41	1.31	1.36	1.36	1.31 (1.24, 1.38)	1.05 (1.00, 1.11)
Suicide death	0.17	0.16	0.19	0.18	0.17	0.18	0.18	0.17	0.18	0.16	0.96 (0.83, 1.11)	1.04 (0.90, 1.20)
Psychiatric hospitalisation	17.8	17.7	17.5	17.2	17.0	16.8	16.7	16.2	16.1	15.8	0.87 (0.85, 0.88)	0.91 (0.89, 0.92)
Emergency department visits	10.3	10.2	10.4	10.0	10.0	10.0	9.8	9.5	9.5	9.8	0.91 (0.89, 0.93)	0.97 (0.95, 0.99)
Employment	36.8	37.3	37.0	36.4	36.4	36.4	36.0	36.5	36.1	36.1	0.96 (0.95, 0.97)	1.05 (1.04, 1.07)
Urban (%)												
All-cause mortality	0.99	1.06	1.08	1.13	1.14	1.19	1.34	1.25	1.30	1.33	1.34 (1.26, 1.44)	1.07 (1.00, 1.14)
Suicide death	0.16	0.18	0.20	0.18	0.17	0.19	0.19	0.17	0.17	0.19	1.01 (0.85, 1.20)	1.10 (0.93, 1.31)
Psychiatric hospitalisation	17.2	17.1	16.9	16.5	16.5	16.2	16.1	15.6	15.5	15.3	0.86 (0.85, 0.88)	0.90 (0.89, 0.92)
Emergency department visits	10.6	10.5	10.7	10.3	10.2	10.2	10.0	9.7	9.6	9.9	0.89 (0.87, 0.91)*	0.95 (0.93, 0.97)*
Employment	34.5	35.3	35.0	34.4	34.5	34.6	34.4	35.0	34.6	34.5	0.99 (0.97, 1.00)*	1.11 (1.09, 1.13)*
Rural (%)												
All-cause mortality	1.17	1.29	1.29	1.30	1.39	1.32	1.56	1.45	1.48	1.43	1.24 (1.12, 1.36)	1.03 (0.93, 1.13)
Suicide death	0.20	0.13	0.16	0.18	0.17	0.17	0.17	0.15	0.21	0.08	0.83 (0.63, 1.09)	0.89 (0.68, 1.18)
Psychiatric hospitalisation	19.0	19.1	18.7	18.6	18.4	18.3	17.9	17.7	17.6	17.0	0.88 (0.85, 0.90)	0.92 (0.89, 0.95)
Emergency department visits	9.6	9.6	9.6	9.1	9.5	9.4	9.2	9.2	9.2	9.4	0.96 (0.92, 1.00)*	1.02 (0.99, 1.06)*
Employment	42.0	42.1	41.8	41.1	40.8	40.6	39.8	40.2	39.9	39.8	0.90 (0.88, 0.92)*	0.95 (0.93, 0.97)*

Adjusted odds ratios were calculated using a multivariate logistic model, which accounted for changes in age groups, gender and urbanicity.

*Significant urban–rural difference (*P* < 0.05).

### Urban–rural difference in quality indicators of healthcare for individuals with schizophrenia

Regarding antipsychotic medication types, SGA and SGA LAI use increased more in rural than urban areas, narrowing the urban–rural gap from 2.1 to 0.9% for SGAs and from 0.8 to 0.4% for SGA LAIs between 2010 and 2019. For adequate daily dose, the gap declined from 10.9 to 9.5%, with higher AORs in rural (1.43; 95% CI: 1.39–1.46) than urban areas (1.31; 95% CI: 1.29–1.33). Medication adherence (MPR ≥ 0.8) decreased in both urban (37.8 to 33.3%) and rural areas (39.0 to 35.7%), with AORs of 0.89 (95% CI: 0.88–0.90) and 0.93 (95% CI: 0.91–0.95), respectively. Benzodiazepine use declined more in rural areas.

However, for outcome indicators, rural–urban patterns were reversed, with greater improvements observed in urban areas. Emergency department visit rates were higher in urban areas, but the gap narrowed from 1.0 to 0.5%. After adjusting for demographic variables, the decline was significant in urban areas (AOR = 0.95, 95% CI: 0.93–0.97) but not in rural areas (AOR = 1.02, 95% CI: 0.99–1.06). In regard to employment, rates were higher in rural areas in 2010 but the absolute gap decreased from 7.5 to 5.3%. The adjusted trend showed an increase in urban areas (AOR = 1.11, 95% CI: 1.09–1.13) and a decrease in rural areas (AOR = 0.95, 95% CI: 0.93–0.97).

## Discussion

### Main finding

Throughout this period, a notable shift in antipsychotic medication usage was observed: a decrease in FGA use alongside increases in that of SGAs and LAIs. The study noted a decline in medication adherence, yet there was an increase in the proportion of individuals receiving an adequate daily dose. Concurrently, there was a decline in anticholinergic and benzodiazepine usage, while the utilisation of antidepressants and mood stabilisers saw an upward trend. Turning to outcome indicators, there were no significant trends in all-cause mortality and suicide rates; however, psychiatric hospitalisations and emergency department visits decreased over the decade. Despite appearances to the contrary, employment rates increased slightly after adjusting for demographic factors. Urban–rural disparities were evident in treatment process indicators, with rural areas showing greater improvement except for medication adherence. Conversely, outcome indicators demonstrated reversed trends in rural–urban comparisons. While urban areas saw a significant decrease in emergency department visits, rural areas did not exhibit statistical significance. Employment rates increased in urban areas but decreased in rural areas.

### Treatment process indicators

We observed an increasing trend in the use of SGAs during the study period, consistent with patterns seen in many countries worldwide.^20^ The increased use of SGAs can be related to their superior efficacy compared with FGAs, and to their association with higher patient adherence and fewer extrapyramidal symptoms.^21^ FGAs are gradually being phased out due to a declining trend in their use.

We also observed a gradual increase in the use of LAIs among individuals with schizophrenia. This observation could potentially be linked to the rise in the number of community psychiatric clinics and the implementation of Schizophrenia P4P healthcare initiatives. Regarding the utilisation of LAIs, Taiwan’s usage aligns closely with the Asian country average, accounting for approximately 16.1% in 2019, compared with the neighbouring countries’ average of around 17.9%.^22^ However, this proportion is significantly lower than that of Singapore and Malaysia, where 40–50% of patients use LAIs.^22^ This finding suggests that there is room for growth in Taiwan and, with policy support, this proportion is expected to continue increasing in the coming years. The variations noted in the use of LAIs in different countries are significant. For example, in Australia, LAIs are up to four times more likely to be used in community treatment compared with oral medications, and the SGA LAI user population tends to be younger, with a shorter duration of illness, less likelihood of experiencing stiffness as a side-effect, but a higher risk of obesity and dizziness, which differs from FGA LAIs.^23^ A French study concluded that the median persistence for SGA LAIs was higher than for oral antipsychotics and FGA LAIs.^24^

It is worth noting that, despite the increasing use of LAIs, we found that the proportion of individuals meeting the MPR ≥ 0.8 adherence threshold actually declined. This discrepancy may be partly due to some LAIs being administered over intervals longer than the recommended schedule, which would artificially lower the calculated MPR. Patients may be more likely to miss scheduled injections when the interval is longer. Another possible explanation is that, as individuals with schizophrenia age or their condition stabilises, physicians might prescribe higher doses of medication, allowing for less frequent clinic visits. As a result, the daily dose increases while the MPR decreases. Considering that outcome indicators concurrently improved, we believe the ‘adequate daily dose’ (DDD ≥ 0.8) can complement MPR as a measure of treatment adequacy in this context. Notably, the proportions of medication adherence and adequate daily dose remained low. Previous research has highlighted that, despite receiving LAIs, many cases still exhibit poor adherence.^15^ Additional strategies are required to enhance treatment adequacy.

The co-prescription ratio of antidepressants and mood stabilisers has been steadily increasing each year. This rise in the use of antidepressants and mood stabilisers may indicate a heightened awareness among psychiatrists regarding mood symptoms in individuals with schizophrenia, prompting more proactive treatment of these symptoms. Research conducted in Finland suggests that the use of antidepressants in individuals with schizophrenia is linked to a reduced suicide mortality rate.^25^ The decline in the use of anticholinergic agents may be related to the increased utilisation of SGAs. It is worth noting that benzodiazepine use remained high among individuals with schizophrenia in Taiwan,^26^ declining only modestly over the decade, with nearly 70% still prescribed these medications in 2019. This rate, higher than in many Western countries, probably reflects local prescribing practices, limited access to psychosocial treatments and historically permissive guidelines.^27^ Although the downward trend is encouraging, further efforts are needed to optimise prescribing, and increased antidepressant use may help reduce reliance on benzodiazepines.

### Outcome indicators

Although the number of deaths has increased over recent years, this may be due to the mean age at which the number of schizophrenia individuals increased. After adjustment for demographic variables, there is no overall change in mortality rates. Although a previous study found an association between LAI use during the early stages of the illness and a decrease in all-cause mortality and suicide,^9^ no significant effect was observed over a 10-year period, possibly because only 16% of patients received LAIs – insufficient to produce a notable impact. Our findings are consistent with a comprehensive 30-year Finnish study which revealed that the all-cause standardised mortality ratio remained consistent throughout the follow-up period.^28^ However, a 20-year cohort study conducted in Denmark unveiled a rising trend in standardised mortality ratios over time, indicating a mean increase of 0.03 per year among individuals diagnosed with schizophrenia.^29^

Interestingly, our study revealed a declining trend in psychiatric hospitalisation rates. While this may partly reflect improvements in schizophrenia care, alternative explanations should be considered. The substantial growth in local psychiatric clinics has allowed more relapses to be managed in out-patient settings. Moreover, the expansion of community-based care may have reduced the need for social admissions, thereby lowering hospitalisation rates. However, this decline was unrelated to the number of psychiatric in-patient beds in Taiwan, which has increased slightly. Of note, the decreasing trend in hospitalisation rates is consistent with a South Korean study, which reported a steady decline in psychiatric hospitalisations from 2015 to 2020.^30^ In contrast, the USA saw a significant increase in hospitalisation numbers from 2005 to 2014, but the average length of hospitalisation was only 9.08 days.^31^ These variances could be related to disparities in healthcare systems and the affordability of medical costs.

After adjustment for demographic shifts, employment rates showed a statistically significant but modest upward trend. Although this trend might be related to changes in psychiatric services, factors beyond the healthcare system probably influenced these patterns, including Taiwan’s macroeconomic conditions – unemployment rates declined from 5.2% in 2010 to 3.7% in 2019^32^ – and nationwide social welfare and disability policies. Notably, the Occupational Reconstruction Service for people with disabilities,^33^ launched in 2009, introduced vocational rehabilitation case managers to coordinate services, integrate local vocational rehabilitation resources and provide continuous professional support throughout the rehabilitation process. These external factors, alongside treatment quality, collectively shape employment outcomes.

### Urban–rural difference

In general, treatment indicators were more favourable in urban compared with rural areas; however, the trend of improvement was more pronounced in the latter. This observation might be associated with increased psychiatric services and the implementation of the Schizophrenia P4P programme, which mitigated the urban–rural disparity. By rewarding facilities for meeting performance benchmarks (e.g. medication management, regular visits) and re-engaging low-frequency patients, the programme promotes active outreach and continuous care, enabling rural areas to expand community services. Evaluations have reported benefits such as fewer compulsory admissions and unscheduled visits,^12^ although not all outcomes improved. Except for medication adherence, rural areas had greater scope for improvement due to their initially lower indicators, but overall treatment indicators remained inferior in rural regions.

Regarding outcome indicators, all-cause mortality rates were higher in rural areas compared with urban; the trends in mortality did not significantly differ between these areas. Health utilisation patterns revealed higher psychiatric hospitalisation rates but lower rates of emergency department visits in rural areas. This discrepancy may indicate a shortage of emergency psychiatric services in rural regions, potentially leading to increased emergency department utilisation and reduced hospitalisation necessity. Moreover, these differences in both outcome and treatment indicators are due to rural communities generally having lower provider density^34^ and limited availability of psychiatric services,^35^ as well as to different socioeconomic challenges, compared with urban communities.

We observed divergent employment trends among individuals with schizophrenia in urban and rural areas. This disparity could be related to differences in economic and social environments, even though welfare and benefit policies are consistent across Taiwan. Factors such as job types, family financial support and local economic conditions might explain the higher employment rates in rural areas. However, it is crucial to note that the overall employment rate decreased in rural areas while it increased in the urban setting. The shift from traditional agricultural roles to service sector jobs, small-scale entrepreneurship and emerging industries may have contributed to this decline in rural employment.^36^ Additionally, the superior treatment quality in urban areas probably played a role in boosting employment rates there, underscoring the potential impact of treatment adequacy on employment outcomes.

### Limitations

This study has some limitations that should be considered when interpreting the results. First, the quality indicators were measured based on the administration database; the actual treatment quality was unknown. Therefore, we could measure only the surrogate indicators of treatment response, such as the rate of psychiatric hospitalisation or employment rate. Second, only a few studies demonstrated the association of these treatment process indicators with clinical outcomes; whether or not these indicators reflect the true quality of care remains unclear. Further studies need to validate these indications. Third, we cannot infer a causal relationship between the changes in psychiatric services and improvements in our observed indicators. Future studies should employ quasi-experimental designs (e.g. interrupted time series or difference-in-differences analyses) to more rigorously test whether initiatives such as the P4P programme directly lead to improved outcomes. Fourth, our analysis ended in 2019 and does not capture potential changes in schizophrenia care during the COVID-19 pandemic. Because healthcare utilisation and treatment patterns may have shifted substantially since 2020, our findings reflect the pre-pandemic period and should be extrapolated with caution. Finally, because this study was conducted within a single national health system, the observed trends may not be fully generalisable to other countries or healthcare settings with different systems of care.

In conclusion, our study highlights significant trends in mental health treatment in Taiwan over the past decade, including a shift towards the use of SGAs and LAIs. We noted reductions in psychiatric hospitalisations and emergency department visits, alongside increases in employment rates, with differences between urban and rural areas. The quality of healthcare for individuals with schizophrenia has improved in tandem with changes in psychiatric services. Further enhancements to the P4P programme – such as targeted health promotion for hard-to-engage patients, additional incentives for community outreach and functional recovery metrics – could strengthen treatment quality and outcomes. Nonetheless, our findings do not establish causality. Future research should evaluate the effects of psychiatric service policies and regularly monitor healthcare quality to further improve the lives of individuals with schizophrenia.

## Data Availability

The data supporting the findings of this study are not available for public access due to ethical restrictions and privacy concerns.
